# Purified PEGylated human glucagon-like peptide-2 reduces the severity of irradiation-induced acute radiation enteritis in rats

**DOI:** 10.1093/jrr/rry076

**Published:** 2018-09-22

**Authors:** Tian Zhang, Lei Shi, Yuan Xu, Yang Li, Shicao Li, Bo Guan, Zhihua Qi, Ye Zhang, Linna Liu

**Affiliations:** 1 Department of Pharmacy, Tangdu Hospital, Fourth Military Medical University, Xi’an, PR China; 2 Department of Infectious Diseases, Tangdu Hospital, Fourth Military Medical University, Xi’an, PR China

**Keywords:** acute radiation enteritis, glucagon-like peptide-2, polyethyleneglycol: peptides: NF-κB

## Abstract

Radiation-induced acute intestinal injury after abdominal and pelvic irradiation is a common and serious problem in the clinical setting. Glucagon-like peptide-2 (GLP-2), a 33-amino acid peptide, exerts diverse effects related to the regulation of gastrointestinal growth and function. However, GLP-2 is relatively unstable *in vivo*. The aim of the present study was to improve GLP-2 stability *in vivo* and to evaluate its therapeutic effect on acute radiation enteritis. We generated long-lasting intestinal protection peptides by conjugating human GLP-2 (hGLP-2) peptides to polyethyleneglycol (PEG) to produce mPEGylation hGLP-2 (Mono-PEG-hGLP-2) through an enzymatic site-specific transglutamination reaction. Mono-PEG-hGLP-2 synthesized under optimal reaction conditions and separated by one-step ion-exchange chromatography was found to be resistant to degradation *in vitro*. Pretreatment with Mono-PEG-hGLP-2 reduced the severity of radiation-induced intestinal injury, oxidative stress, and the expression of NF-κB in rats with irradiation-induced acute radiation enteritis. The enhanced biological potency of Mono-PEG-hGLP-2 highlights its potential as a therapeutic agent for intestinal diseases.

## INTRODUCTION

Radiotherapy is an important aspect of multimodal cancer therapy. Radiation-induced acute intestinal injury after abdominal and pelvic irradiation is a common and serious problem [1–3] that is progressive in nature, and in the clinic, it can result in symptoms such as nausea, vomiting and diarrhea. Radiotherapy can damage intestinal barrier function and induce changes in bacterial flora, vascular permeability of mucosal cells, and intestinal motility [1]. Therefore, the morbidity and mortality due to radiation enterocolitis is high. Complications of abdominopelvic radiotherapy are a major problem for clinicians. These complications are treated with medications, antibiotics, anti-inflammatory drugs, hyperbaric oxygen, or by an interruption of therapy [4, 5].

Glucagon-like peptide-2 (GLP-2) is a 33-amino acid peptide derived from the post-translational processing of proglucagon in enteroendocrine L-cells and cerebral neurons [6]. GLP-2 exerts diverse effects related to the regulation of gastrointestinal growth and function, including stimulation of mucosal growth, inhibition of apoptosis [7, 8], increased nutrient absorption [9], inhibition of gastric emptying and gastric acid secretion [10], reduction of intestinal permeability [11], stimulation of intestinal blood flow [12], and relaxation of intestinal smooth muscle [13]. GLP-2 has promising therapeutic potential in experimental models of intestinal atrophy and injury, including mucosal atrophy [14, 15], short bowel syndrome [16, 17], experimental colitis [18, 19], immune-mediated inflammatory bowel disease [20], and the prevention of chemotherapy-induced apoptosis [21]. However, GLP-2 is relatively unstable *in vivo*. The plasma half-life is 7 min; therefore, clinical application requires frequent administration at high dosages. These limitations pose challenges in the use of GLP-2 for the treatment of intestinal diseases.

The rapid clearance and degradation of GLP-2 in plasma is mainly due to N-terminal cleavage of the first two amino acids, His1-Ala2, by the enzyme dipeptidyl peptidase IV (DPP-IV) [22]. Protein modification is a convenient technique for improving the therapeutic profiles of native proteins. PEGylation slows down drug absorption, which decreases serious side effects caused by acute peak drug concentrations, prolongs a drug’s half-life, and improves patient compliance by reducing the frequency of injections [23, 24]. Qi *et al.* found that PEGylated porcine GLP-2 was resistant to degradation and reduced the severity of colonic injury in murine colitis [24].

In the present study, we generated long-lasting intestinal protection peptides by conjugating hGLP-2 peptides to polyethyleneglycol (PEG) through an enzymatic site-specific transglutamination reaction. Modified hGLP-2 was long-lasting and protected from degradation by the DPP-IV enzyme. PEGylation of hGLP-2 reduced the severity of irradiation-induced acute radiation enteritis in rats.

## MATERIALS AND METHODS

### Materials

The hGLP-2(1–33) amide (>98% purity) was synthesized by GL Biochem Ltd (Shanghai, China). Succinimidyl propionate monomethoxy (PEG) [mPEG-SPA; molecular weight (MW) = 5 kDa] was purchased from Beijing Kaizheng Biotech Development Co., Ltd (Beijing, China). The ion-exchange chromatography (IEC) resin and column used were CM Sepharose Fast Flow (FF), and they were purchased from GE Healthcare Bio-Science AB (Uppsala, Sweden). Ultrafiltration membranes with a MW cut-off value of 3000 were purchased from Millipore Corporation (Billerica, MA, USA). DSS (MW = 36 000–50 000) was purchased from MP Biomedicals China (Shanghai, China). Unless otherwise specified, all of the other chemicals and reagents were of analytical grade from Sigma-Aldrich and Fluka (Milan, Italy).

### PEGylation of pGLP-2 to synthesize mPEGylation hGLP-2 (Mono-PEG-hGLP-2)

The hGLP-2 (MW = 3922.38; 1 mg/ml) peptide in a 50 mmol/l Tris-HCl buffer solution (pH 8.5) was reacted with mPEG5k-SPA (molar ratio of mPEG-SPA to hGLP-2 was set to 1:1, 2:1, 3:1 and 4:1) at 25°C or 37°C for 5 h. The reaction was quenched by adjusting the pH with 1% trifluoroacetic acid (TFA) diluted in deionized water (DW). The PEG-to-peptide molar ratio was assessed.

### Reverse-phase high-performance liquid chromatographic analysis of PEGylation mixtures

The reaction mixture was subjected to reverse-phase (RP) high-performance liquid chromatography (HPLC) on a ZORBAX SB-C_18_ column (4.6 mm × 250 mm, 5 μm; Agilent Technologies, USA) at room temperature. After a 20-μl sample was injected, gradient elution was conducted at a flow rate of 1 ml/min with solvent A (0.1% TFA in DW) and solvent B (0.1% TFA in acetonitrile) using a 38–50% B linear gradient over 15 min. Eluates were monitored at 215 nm.

### Ion-exchange chromatography separation

The PEGylated pGLP-2 mixtures were separated and purified by ion-exchange chromatography (IEC). Cation exchanges (CM Sepharose Fast Flow) were done at pH 4.0 20 mmol/l acetate buffer (equilibration phase) and pH 4.0 20 mmol/l acetate buffer + 1 mol/l NaCl (elution phase). After the PEGylation reaction, the mixture was directly diluted with five times the volume of equilibration buffer (the column was packed with 20 ml of CM Sepharose FF resins) at a flow rate of 1 ml/min. When washing was completed, a continuous gradient from 0% to 100% of elution buffer was applied to elute the PEGylated pGLP-2 products. After each PEGylated product was collected, desalted, and concentrated by the 3-kDa cut-off value, ultrafiltration and lyophilization to powder were conducted before further characterization.

### Mono-PEG-hGLP-2 stability *in vitro*

The hGLP-2 (100 μl, 25 nmol/l) peptide and an equivalent amount of purified mono-PEG-hGLP-2 were prepared in triethylamine·HCl buffer (10 mmol/l, pH 7.4). DPP-IV (100 mU/ml, 900 μl) was added, and the solutions were incubated at 37°C. At the indicated time points, 100 μl was removed from the reaction mixture, and reactions were terminated by adding 10 μl of 10% (v/v) TFA. Each sample was analyzed by RP-HPLC as described.

### Animal studies

#### Rats and maintenance

Sixty adult male Sprague Dawley (SD) rats weighing 220–250 g were included in our study. Rats were subjected to 1 week of preliminary conditioning, during which time they received standard pellet rat chow and water *ad libitum*. They were housed in a temperature-and humidity-controlled environment, two animals per cage, with a 12-h light/12-h dark cycle. The Principles of Laboratory Animal Care (NIH Publication 85e23, revised 1996) were followed. Animal study protocols were approved by the Ethics Committee of the Fourth Military Medical University. The experiment was carried out at the Department of Pharmacy, Tangdu Hospital, Fourth Military Medical University.

#### X-ray irradiation

X-ray irradiation was performed using a linear accelerator (Siemens primask) adjusted to provide X-ray irradiation, and carried out at the Department of Radiology and Oncology at the Tangdu Hospital. All of the animals were anesthetized with an intramuscular injection of pentobarbital sodium (100 mg/kg) and subjected to a single dose of 8 Gy abdominal X-radiation. The dose was delivered at a rate of 400 monitor units/min (1100 rad, 116.5 cGy/min, 100 cm source–skin distance) using a conventional radiation source, while the other parts of the body were carefully shielded [25, 26].

#### Experimental design

Rats were randomized into six groups (Groups A–F). The control group (Group A) did not receive radiation and received only standard rat chow and water every day. The remaining rats were subjected to a single dose of 8 Gy of abdominopelvic X-radiation and randomized further into five groups (Groups B–F). This dose has been shown to reliably produce radiation-induced enteritis in rats [25]. Briefly, rats were anesthetized with 100 mg/kg pentobarbital sodium, positioned supine, and subjected to a single fraction of 8 Gy delivered at a specific depth through abdominopelvic fields using X-rays at the rate of 0.74 Gy/min, while the other parts of the body were carefully shielded. Radiated rats were allowed to recover from the anesthetic and GLP-2 or Mono-PEG-hGLP-2 was administered by intraperitoneal injection.

Group A: non-X-ray irradiated (control).
Group B: X-ray irradiated.
Group C: pretreated with 3 μg of Mono-PEG-hGLP-2 in 0.3 ml of saline 5 min before X-ray irradiation.
Group D: pretreated with 10 μg of Mono-PEG-hGLP-2 in 0.3 ml of saline 5 min before X-ray irradiation.
Group E: pretreated with 30 μg of Mono-PEG-hGLP-2 in 0.3 ml of saline 5 min before X-ray irradiation.
Group F: pretreated with 30 μg of GLP-2 in 0.3 ml of saline 5 min before X-ray irradiation.

#### Abdominal laparotomy and morphological examination of the small intestine

Animals were killed 48 h after X-ray irradiation, and laparotomies were performed. Immediately after the laparotomy, the small intestine (jejunum) was examined and removed. Two-centimeter segments of the jejunum and segments of the intestine showing edema and change in color (purple) were resected. Luminal contents were washed with 0.9% NaCl by injecting saline through the lumen of the small bowel segments. Tissue samples were pinned out on a paraffin block and floated upside down in 10% buffered formalin overnight, dehydrated, and embedded in paraffin. Sections of 4 μm were cut with a Leica sliding microtome (SM 2000R, Nussbach, Germany) and slides, containing a minimum of 10 sections, were stained with hematoxylin and erosin stains. Some representative intestine sections were processed for ultrastructural studies. Sections were evaluated quantitatively and qualitatively in a blinded fashion [25].

#### Histological evaluation of the small intestine

For histological assessment, villous height (from the base to the tip of the villi), crypt height (from the base to the tip of each crypt), and the number of villi per square millimeter was determined based on individual intestinal segments at five separate microscopic fields for each animal. They were recorded as the mean values using ocular micrometer-adapted light microscopy at a magnification of ×100.

#### Biochemical measurements

Malondialdehyde (MDA) levels, glutathione (GSH) content, and the activities of super oxide-dismutase (SOD) and glutathione peroxidase (GPx) were determined in the small intestine using commercial assay kits (Biyuntian Co., China) according to the manufacturer’s protocols. The protein content ratio was determined by Pierce™ BCA Protein Assay Kit (Thermo Fisher Scientific Inc., Rockford, IL, USA). All of the values were normalized to total intestinal protein. Small intestine tissue (20 mg) was homogenized in lysis buffer (150 mM NaCl, 0.1% SDS, 0.1% NP-40, 20 mM Tris-HCl pH 7.5) and protease inhibitor cocktail using a Tissue Lyser II homogenizer (Qiagen; Hilden, Germany). Total protein concentrations of tissue homogenates were detected by a Pierce™ BCA Protein Assay Kit (Thermo Scientific; Rockford, IL, USA). The levels of tumor necrosis factor-alpha (TNF-α) (Invitrogen; Carlsbad, CA, USA) and interleukin-2 (IL-2) (Bioo Scientific; Austin, TX, USA) in the tissue samples were determined using commercially available sandwich ELISA kits, according to the protocol recommended by the manufacturer. Results were recorded as the ratio of detected protein (TNF-α, IL-2) (pg/ml) to the amount of total protein (mg/ml).

#### Immunohistochemistry

Rat tissues were perfused with phosphate-buffered saline (PBS), sliced, and fixed with 4% paraformaldehyde for the immunohistochemical experiments. Intestinal samples were incubated overnight in PBS with 6.8% sucrose, dehydrated with acetone, and paraffin-embedded. Before staining, semi-thin sections were incubated for 5 min at 37°C in 0.01% trypsin/0.1% CaCl_2_ (pH 7.8). Sections were incubated for 5 h at 37°C with NF-κB antibodies (Abcam, UK), and then sections were again rinsed three times in 0.1 M PBS for 5 min each, incubated with secondary antibody for 15–20 min, rinsed three times in 0.1 M PBS for 5 min each, incubated at 37°C for 30 min, and rinsed four times in 0.1 M PBS for 5 min each. Diaminobenzidine (DAB) was applied for 3–5 min until a brown-colored reaction product was observed. After rinsing the sections in distilled water three times, the sections were counterstained with hematoxylin for 10 s, rinsed three times in distilled water, and dehydrated with an ascending concentration (i.e. 80%, 90%, 95% and 100%) of ethanol for 10 min each. Sections were then cleaned and mounted and coverslips were added for immunohistochemical study. Images were captured with a Nikon photo microscope equipped with a digital camera.

#### Immunoblot analysis

The expression of NF-κB was analyzed by Western blotting. We separated 50 μg protein on 6.25% sodium dodecyl sulfate–polyacrylamide gels (SDS–PAGE) using the mini-PROTEAN 3 electrophoresis cell (Bio-Rad Laboratories, Inc., Hercules, California, USA) according to the manufacturer’s protocol. After being transferred to a nitrocellulose membrane, the proteins were blocked for 1 h at room temperature with 5% non-fat dry milk in Tris-buffered saline containing 0.05% Tween 20 (TBST). The membrane was then incubated with anti- NF-κB antibody for 1 h, washed four times with TBST, and incubated for 1 h with a peroxidase-conjugated secondary antibody (1:1000). After washing the membrane four times with TBST buffer, the blot was developed using ECL chemiluminescence reagents (Millipore immobilon^TM^ HRP Substrate) and imaged. The immunoreactive bands on the autoradiography films were scanned with a calibrated densitometer ChemiDoc^TM^ XRS+ (Bio–Rad imaging System) and quantified using QuantityOne imaging software (Bio–Rad Laboratories, Hercules, CA). Equal loading of proteins onto the gel was confirmed by the immunodetection of GAPDH.

### Data analysis

Data are expressed as the mean ± SD. Statistical differences between groups were determined by ANOVA. The level of significance were considered as follows: *P* > 0.05, non-significant; *P* < 0.05, significant; and *P* < 0.01, highly significant. Computations were performed with SPSS version 11.0 software. All of the analysis was performed in a blinded fashion by the authors.

## RESULTS

### Preparation and purification of Mono-PEG-hGLP-2

#### Determining optimal molar ratios of mPEG-SPA to pGLP-2

**Fig. 1.**
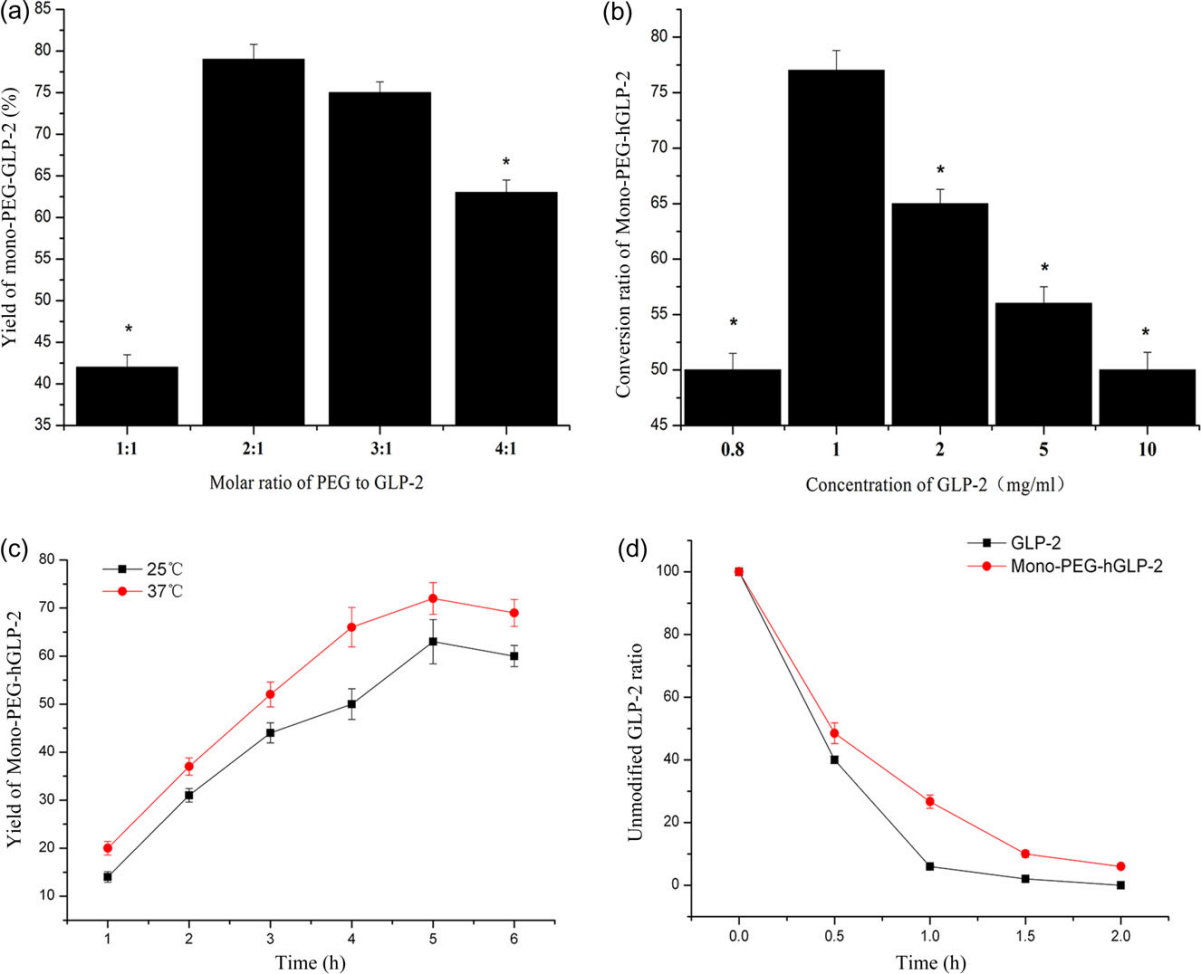
(a) Mono-PEG-hGLP-2 yield in different molar ratios of mPEG-SPA to pGLP-2, ^*^P < 0.05 vs the molar ratio of mPEG-SPA to pGLP-2, 2:1. (b) The conversion ratio of Mono-PEG-hGLP-2 in different concentrations of GLP-2, ^*^P < 0.05 vs the concentration of GLP-2 in 1 mg/ml. (c) Influence of reaction temperature and reaction time on the yield of Mono-PEG-hGLP-2. (d) Proteolytic stability of Mono-PEG-hGLP-2 and GLP-2.

To determine the optimal reaction conditions for PEGylation, the feed molar ratios of mPEG-SPA to pGLP-2 was set to 1:1, 2:1, 3:1 and 4:1 at different time intervals by HPLC (Fig. 1a). Increased ratios of mPEG-SPA to pGLP-2 augmented the yield of Mono-PEG-hGLP-2, but the yield was not increased until it reached a certain level. Increases in the molar ratio of mPEG-SPA resulted in multiple modification products; thus, the conversion ratio of Mono-PEG-hGLP-2 decreased. The optimal ratio of mPEG-SPA to pGLP-2 was 2:1.

#### Influence of GLP-2 concentration on Mono-PEG-hGLP-2 production

In the process of modification, the concentration of the GLP-2 had a great effect on the conversion ratio of Mono-PEG-hGLP-2. At lower concentrations of GLP-2, the conversion ratio of Mono-PEG-hGLP-2 rose as the concentration of GLP-2 rose (Fig. 1b). When the concentration was >1 mg/ml, the conversion ratio of Mono-PEG-hGLP-2 declined as the concentration of GLP-2 rose. At higher concentrations, multiple modifications can occur, resulting in a decrease in Mono-PEG-hGLP-2 and an increase in GLP-2 concentrations. The optimal concentration of GLP-2 was determined to be 1 mg/ml.

#### Influence of reaction temperature and reaction time on the yield of Mono-PEG-hGLP-2

The effect of temperature on the yield of Mono-PEG-hGLP-2 was measured in reactions containing a 2:1 ratio of mPEG-SPA to pGLP-2 and 1 mg/ml GLP-2. The yield of Mono-PEG-hGLP-2 increased with the reaction temperature (Fig. 1c). After reaching a certain temperature, the optical activity of chiral amino acid was easy to change, thus the activity of hGLP-2 decreased. The optimal reaction temperature and reaction time were 37°C and 5 h, respectively.

#### Isolation and purification of Mono-PEG-hGLP-2

**Fig. 2.**
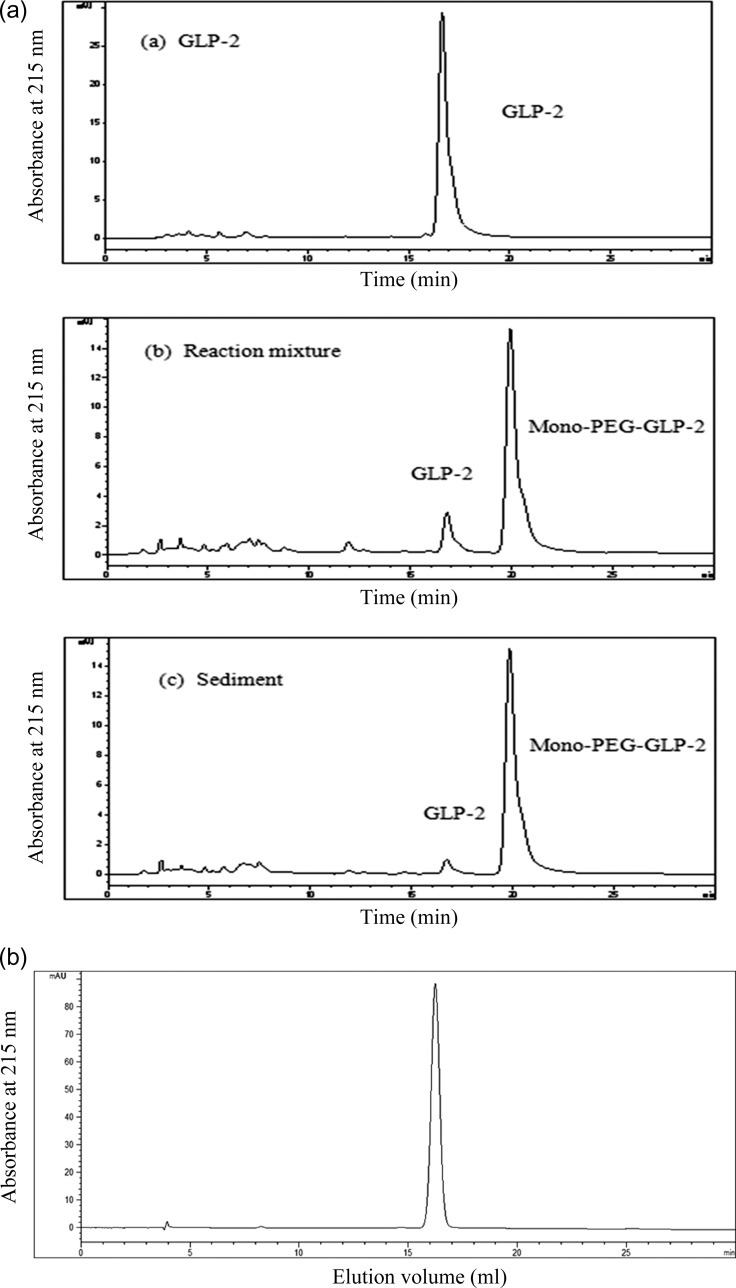
(a) Reverse-phase high-performance liquid chromatography profiles of GLP-2, reaction mixture, and sediment of reaction; (b) high-performance gel filtration liquid chromatography of purified Mono-PEG-GLP-2 by reverse-phase high-performance liquid chromatography. The purity of Mono-PEG-GLP-2 was 97% and the yield was 95%.

After the PEGylation reaction, a small amount of unmodified GLP-2, multi-PEG-hGLP-2, and unreacted mPEG-SPA were present. The PEGylated product was isolated by cation exchange chromatography (SP-Sepharose Fast Flow), desalted by ultrafiltration, and freeze-dried. Cryogenic centrifugation was introduced to separate the Mono-PEG-hGLP-2. The reaction liquid was centrifuged at 10 000 rpm under 4°C for 15 min, then, the supernatant and sediment were detected. As shown in Fig. 2a and Table 1, refrigerated centrifugation removed most of the unmodified GLP-2. Sediment was dissolved with water and reverse-phase liquid chromatography was used to purify the product. The product purity was 97% and the yield was 95% (Fig. 2b).

**Table 1.** Mono-PEG-hGLP-2 and GLP-2 concentration in the reaction mixture, sediment, and supernatant

Group	Reaction mixture	Sediment	Supernatant
GLP-2 (%, w)	17	3.6	92
Mono-PEG-GLP-2 (%, w)	83	95.8	6

#### Proteolytic stability of Mono-PEG-hGLP-2

Unmodified GLP-2 does not have a complex secondary structure, so it is easily degraded by proteolytic enzymes. After 1 h of enzymatic hydrolysis, 26.7% of Mono-PEG-hGLP-2 remained, while only 5% unmodified GLP-2 was present (Fig. 1d). These results demonstrate that the modification of GLP-2 increases the resistance to protease hydrolysis.

### Effects of Mono-PEG-hGLP-2 on radiation-induced intestinal injury in rats

#### Histological and morphometric changes

**Fig. 3.**
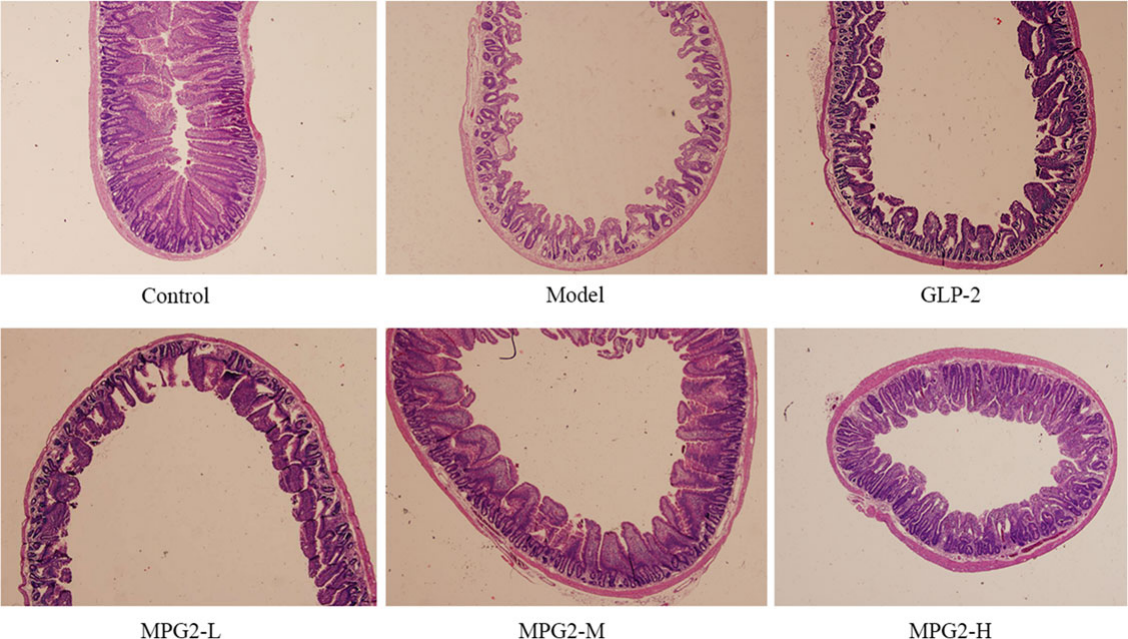
Histological and morphometric changes in small intestinal villi of the rats. MPG2-L: pretreated with 3 μg of Mono-PEG-hGLP-2. MPG2-M: pretreated with 10 μg of Mono-PEG-hGLP-2. MPG2-H: pretreated with 30 μg of Mono-PEG-hGLP-2.

All of the rats tolerated the experiments and survived throughout the duration of the study. Mono-PEG-hGLP-2 pretreatment markedly protected the integrity of the villi and preserved the villous height (Fig. 3). Irradiated mice had a significantly reduced number and height of small intestine villi in comparison with the control group (Table 2) (group model, *P < 0.01*). However, the height of the villi decreased slightly in the Mono-PEG-hGLP-2 pretreatment group. The villi height in mice pretreated with high doses of Mono-PEG-hGLP-2 was not significantly different from the control group (*P < 0.05*). Unmodified GLP-2 pretreatment did not protect mice against radiation-induced intestinal injury.

**Table 2.** Clinical pathological and morphometric changes in rats

Animal findings	Control (non-X-ray irradiation)	X-ray irradiated model group	MPG2–L pretreatment	MPG2–M pretreatment	MPG2–H pretreatment	GLP-2 pretreatment
**Clinical**						
Diarrhea	–	+	–	–	–	+
Abdominal distention	–	+	+	–	–	+
**Gross**						
Intestinal dilatation	–	+	+	–	–	+
Mucosal ulceration	–	+	+	–	–	+
Edema	–	+	+	+	–	+
**Histologic**						
Shortening of villi	–	+	+	+	–	+
Architectural disarray	–	+	+	–	–	+
**Morphometric**						
Number of villi (mm^2^)	53.2 ± 3.7	28.7 ± 2.9**	32.1 ± 3.3	36.7 ± 2.9*	43.1 ± 4.1*	32.1 ± 4.1**
Height of villi (μm)	543.1 ± 26.8	233.1 ± 14.8**	268.1 ± 11.6*	437.1 ± 19.4*	507.9 ± 21.1*	229.7 ± 15.9**
Crypt height (μm)	219.5 ± 10.8	104.6 ± 8.8**	121.6 ± 6.3*	104.6 ± 8.8*	188.5 ± 8.3*	113.8 ± 10.9**

(–) = absent, (+) = present, MPG2 L–H = pretreatment with Mono-PEG-hGLP-2 (MPG2) in low, middle or high dosages. ^**^P < 0.01, compared with the control group. ^*^P < 0.05, MPG2 pretreatment compared with the model group.

#### Mono-PEG-hGLP-2 had a significant effect on intestinal antioxidant enzymes and inflammatory factors

Exposure to X-rays significantly increased MDA concentrations, but decreased the concentration of GSH, when compared with the control rats (*P < 0.01*), suggesting that lipid peroxidation was induced (Table 3). At various doses, Mono-PEG-hGLP-2 treatment decreased MDA concentration and restored the GSH level in X-ray–treated rats. Intestinal SOD and GSH-Px activities in the treated group were significantly lower than in the control group (Table 3). Pretreatment with Mono-PEG-hGLP-2 enhanced the activities of antioxidant enzymes in the intestinal lumen (Table 3). Mono-PEG-hGLP-2 also significantly reduced the level of TNF-α and IL-2 in comparison with the levels in the untreated rats. In contrast, unmodified GLP-2 had no significant protective effect on the X-ray–treated rats.

**Table 3.** Intestinal contents of MDA, SOD, GSH, Gpx, and TNF-α and IL-2

Animal groups	Control group	X-ray group	MPG2-L group (C)	MPG2-M group (D)	MPG2-H group (E)	GLP-2 Group (F)
MDA (mmol/mg prot)	3.58 ± 0.09	5.79 ± 0.34**	5.27 ± 0.44	4.48 ± 0.37*	4.01 ± 0.22*	5.51 ± 0.47**
SOD (U/mg prot)	67.53 ± 8.76	33.27 ± 5.68**	40.47 ± 5.41*	47.45 ± 8.24*	56.11 ± 6.01*	35.27 ± 4.66**
GSH (mg/g prot)	33.51 ± 6.12	17.02 ± 4.27**	20.22 ± 4.87	24.70 ± 10.22	24.01 ± 4.17*	18.22 ± 3.44**
GSH-Px (U/mg prot)	478.5 ± 31.2	267.5 ± 41.3**	331.2 ± 22.1*	367.5 ± 42.3*	412.7 ± 65.7*	246.1 ± 37.8**
TNF-α (pg/mg prot)	3.52 ± 0.45	10.21 ± 1.21**	7.68 ± 0.77*	6.12 ± 1.01*	4.78 ± 0.67*	9.78 ± 1.06**
IL-2 (pg/mg prot)	0.45 ± 0.08	1.12 ± 0.11**	1.01 ± 0.13	0.78 ± 0.09*	0.66 ± 0.11*	1.24 ± 0.13**

^**^P < 0.01versus control group, ^*^P < 0.05 versus X-ray group. Data are reported as mean ± S.D. (*n* = 10 in each group). MPG2 L–H = pretreatment with Mono-PEG-hGLP-2 (MPG2) in low, middle or high dosages).

#### Mono-PEG-hGLP-2 significantly decreased NF-κB expression in the intestine

**Fig. 4.**
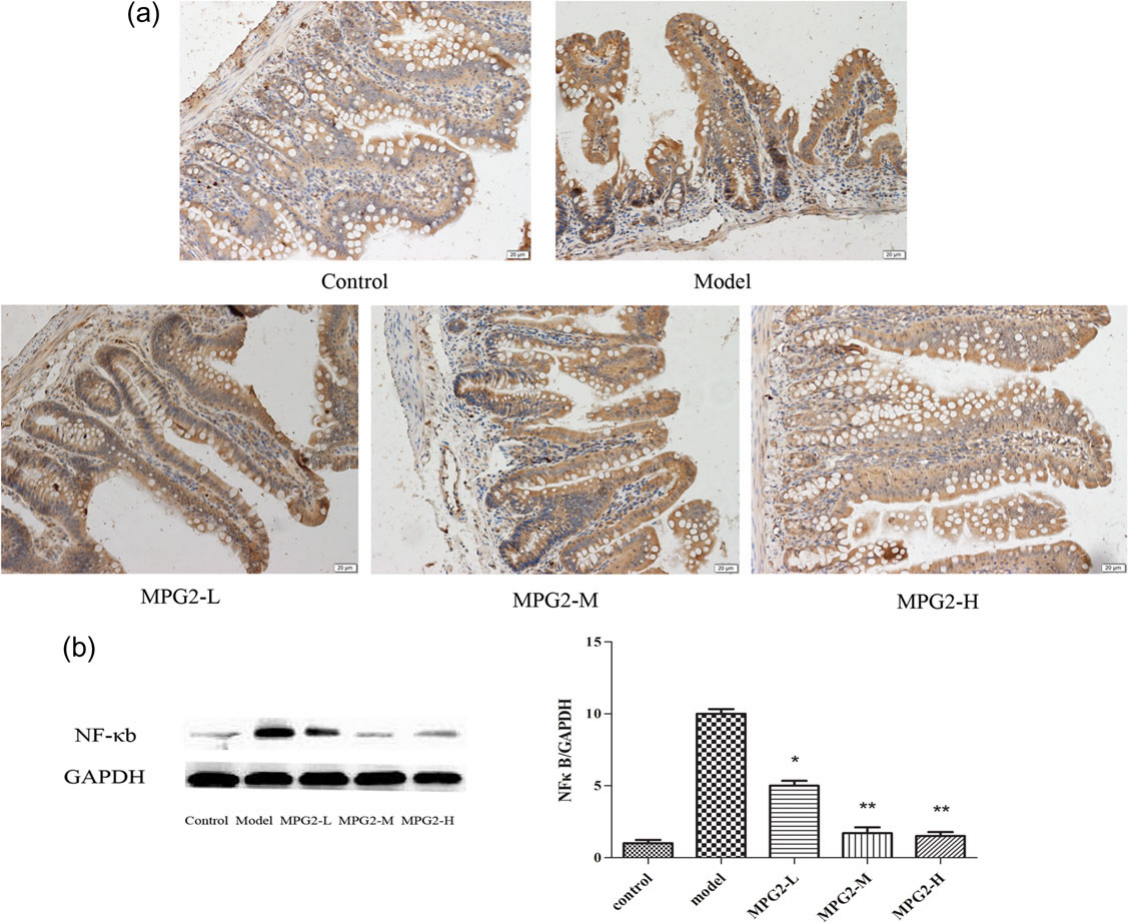
Immunohistochemical (a) and Western blot analysis (b) of NF-κB in intestines. MPG2-L: pretreated with 3 μg of Mono-PEG-hGLP-2; MPG2-M: pretreated with 10 μg of Mono-PEG-hGLP-2; MPG2-H: pretreated with 30 μg of Mono-PEG-hGLP-2, *P < 0.05, **P < 0.01 vs model.

NF-κB increased in the X-ray–treated rats in comparison with the control rats as assessed by immunohistological staining (Fig. 4). Pretreatment with Mono-PEG-hGLP-2 decreased NF-κB staining in a dose-dependent manner in comparison with the X-ray-alone–treated rats. As demonstrated in Fig. 4, moderate to high doses of Mono-PEG-hGLP-2 pretreatment significantly inhibited NF-κB expression (*P* < 0.01).

## DISCUSSION

PEGylation improves drug solubility and decreases immunogenicity through the addition of a non-toxic, non-immunogenic polymer to peptides. PEGylation also increases drug stability and the retention time of conjugates in the blood, and reduces proteolysis and renal excretion, thereby allowing for reduced dosing frequency [23, 24]. The optimal synthesis of Mono-PEG-hGLP-2 was dependent on the molar ratios of mPEG-SPA to pGLP-2, temperature, and time of reaction. The optimal ratio of mPEG-SPA to pGLP-2 was 2:1. Increasing the molar ratio of mPEG-SPA to pGLP-2 resulted in the increased production of multi-modification products. Peptides are sensitive to heat; therefore, the reaction temperature must be controlled to preserve peptide bioactivity and heterogeneity. The optimal reaction temperature was 37°C. Additionally, the concentration of GLP-2 in the synthesis reaction was important, as low concentrations did not produce sufficient Mono-PEG-hGLP-2, while high concentrations resulted in increased byproducts. The best concentration of GLP-2 was 1 mg/ml.

Radiation-induced acute intestinal injury after abdominal and pelvic irradiation is a common and serious problem in the clinical setting. An estimated 5–15% of patients who receive radiotherapy develop complications, such as abdominal pain, diarrhea, and fluid loss [1]. Radiation-induced acute intestinal injury is clinically managed through the treatment of symptoms. Safe and effective approaches for preventing or alleviating radiation-associated enteritis are lacking. In this study, we used 8 Gy of X-radiation as the experimental dose, mainly based on clinical symptoms: model rats showed significant symptoms of diarrhea, abdominal distention, intestinal dilatation, mucosal ulceration and edema. When choosing 12 Gy, the mortality rate of rats was too high, so we finally used 8 Gy as the experimental dose. To quantify the level of protection provided by Mono-PEG-hGLP-2 pretreatment, we measured villi height and number. Villi epithelial cells renew constantly, resulting in the repair of an injury within 24 h; therefore, the detection of the height and width of villi can be helpful to judge the degree of damage to the intestinal mucosa and its repair and proliferative ability [18]. We found that Mono-PEG-hGLP-2 pretreatment significantly increased the number and the height of small intestinal mucosal villi in comparison with the X-ray-only–treated rats.

For protection against X-ray–induced damage the activity of antioxidants and the inhibition of free radicals is important [27]. Antioxidant defense systems play important roles in preventing damage caused by oxygen-free radicals, like ROS. The change in antioxidant enzyme activities is relevant to the ability of the intestinal to cope with oxidative stress during X-ray irradiation [27–29]. The SOD enzyme catalyzes the reduction of the superoxide radical to hydrogen peroxide, keeping the intracellular concentrations of the superoxide radical low. We tested whether pretreatment with Mono-PEG-hGLP-2 affected levels of SOD or GSH, or GSH-Px activity. Furthermore, we assessed MDA levels, an indicator of oxidative liver injury. Levels of SOD and GSH, and GSH-Px activity were significantly lower in the X-ray–treated rats in comparison with those in the control rats, while MDA levels were significantly higher. Pretreatment with Mono-PEG-hGLP-2 significantly changed MDA, SOD, GSH and GSH-Px levels. The results indicated that Mono-PEG-hGLP-2 affected antioxidant activity and protected rats from X-ray–induced oxidative intestinal damage.

Radiation activates inflammatory cells, leading to the synthesis and release of cytokines and inflammatory mediators, such as TNF-α and IL-2 [30]. Biochemical tests showed that pretreatment with Mono-PEG-hGLP-2 significantly reduced the levels of TNF-α and IL-2 compared with the levels in X-ray–treated control rats. During the inflammatory process, NF-κB is activated by a wide variety of agents, including hydrogen peroxide, ozone, and reactive oxygen intermediates. Once activated, NF-κB transcriptionally regulates many cellular genes implicated in early immune, acute phase, and inflammatory responses. The amount of activated NF-κB correlates with the degree of mucosal inflammation [31]. The activation of NF-κB is thought to be part of a stress response because it is activated by a variety of stimuli that include growth factors, cytokines, lymphokines, UV, pharmacological agents, and stress. However, NF-κB not only stimulates the release of inflammatory factors, and promotes the occurrence of inflammatory reactions, but it also plays a protective role in the intestine cells. The activation of NF-κB is the regulating mechanism of the body’s response external stimuli. In this study, we found that Mono-PEG-hGLP-2 influenced cytokine-mediated inflammation and was a potent NF-κB signal inhibitor that decreased TNF-α and IL-2 production. MPEG-GLP-2 were able to downregulate the protein expression of NF-κB, but the mechanism is not yet clear. MPEG-GLP-2 was shown to be a direct inhibitor of NF-κB, decreasing the release of inflammatory factors, and thus reducing intestinal damage, or having a protective effect through other mechanisms that reduce the stimulation of NF-κB and its expression; this needs further research. Our data suggests that Mono-PEG-hGLP-2 pretreatment could antagonize intestinal mucosa injury caused by radiation and promote the proliferation and repair ability of intestinal mucosa.

## CONCLUSION

Mono-PEG-hGLP-2 was found to be resistant to degradation *in vitro*, and it was able to reduce the severity of radiation-induced intestinal injury in rats. These findings may add a new perspective to the potential therapeutic use of Mono-PEG-hGLP-2 for acute radiation enteritis.
